# Responsiveness of eyes with polypoidal choroidal vasculopathy with choroidal hyperpermeability to intravitreal ranibizumab

**DOI:** 10.1186/1471-2415-13-43

**Published:** 2013-08-20

**Authors:** Shozo Sonoda, Taiji Sakamoto, Hiroki Otsuka, Narimasa Yoshinaga, Toshifumi Yamashita, Yuya Ki-I, Akiko Okubo, Takehiro Yamashita, Noboru Arimura

**Affiliations:** 1 Department of Ophthalmology, Kagoshima University Graduate School of Medical and Dental Sciences, Kagoshima, Japan

**Keywords:** AMD, Drug, Enhanced depth imaging optical coherence tomography interventional immunology, Ranibizumab

## Abstract

**Background:**

To determine the role played by vascular endothelial growth factor (VEGF) in polypoidal choroidal vasculopathy (PCV) based on an interventional immunology theory.

**Methods:**

Eyes with PCV were divided in a masked fashion into those with choroidal hyperpermeability (HP group) and those with normal choroidal permeability (NP group) based on the indocyanine green angiograms. The inter-rater agreement rate was evaluated using Fleiss’ kappa. Patients were treated by intravitreal ranibizumab (IVB). The central choroidal thickness and central foveal thickness (CFT) at the baseline and 7 days after the treatment were measured by optical coherence tomography.

**Results:**

Among the 57 consecutive eyes diagnosed with PCV, 42 eyes of 42 patients met the inclusion criteria (21 eyes/HP group vs 21 eyes /NP group). Central choroidal thickness in HP group was significantly thicker than that in the NP group (*P* < .001, Mann–Whitney U test). The inter-rater agreement was high with a Fleiss’ kappa = 0.95, *P* < .0001. The percentage reduction in the CFT in HP group (14.0%) was significantly less than that in NP group (20.4%; *P* = .013, Mann–Whitney U test).

**Conclusions:**

Eyes with PCV that are associated with choroidal hyper-permeability may not be strongly associated with VEGF-related pathology, and may not respond favorably to anti-VEGF monotherapy.

## Background

Polypoidal choroidal vasculopathy (PCV) is characterized by the presence of polyp-like lesions at the terminals of a branching network of choroidal vessels and is the more common type of exudative age-related macular degeneration (AMD) in the Asian population [1,2]. Although typical AMD and PCV are placed under the same category of exudative AMD [3], some cases of PCV have been reported to have a unique characteristic of choroidal vascular hyperpermeability [4,5]. This may indicate that this subgroup of PCV eyes with hyperpermeability has properties that are more like those of eyes with central serous chorioretinopathy (CSC) which also have vascular hyperpermeability.

The major treatments for a subfoveal PCV are photodynamic therapy (PDT), intravitreal ranibizumab (IVR), an anti-vascular endothelial growth factor (VEGF) antibody, or a combination of PDT and anti-VEGF therapy [1,6-10]. The efficacy of IVR for PCV has been reported to be less than that for typical AMD [6,11,12]. Thus, a more effective and safer treatment is needed [12].

The rationale for IVR is that it targets and deactivates VEGF, and its effectiveness has been generally determined by a reduction in retinal thickness. Thus, a measurement of choroidal thickness may provide clues that might help determining not only the effectiveness of IVR but also help determine the pathogenesis of PCV [13,14]. For example, it was reported that the choroidal thickness is related to the AMD subtype, choroidal hyperpermeability, and gene polymorphisms [15-17].

Biologic agents can facilitate the dissection of the role played by the target of the agent in the disease process. Consequently, disease-focused investigations can acquire a level of experimental sophistication that has been the exclusive domain of animal studies [18]. Not only can this form of experimental medicine add substantially to our understanding of the pathogenesis of disease, but it can also aide in the development of new therapies. This concept has been called interventional immunology which is now a fast growing field in experimental medicine [19]. Similarly, treatments based on antibody-drug methodology, e.g., IVR, can produce a so-called knock-out (KO) condition in human eyes [20]. This would then provide direct evidence on the effects of VEGF on the disease process.

Thus, the purpose of this study was to determine the responsiveness of eyes with PCV to IVR. To accomplish this, we treated eyes with PCV and choroidal hyperpermeability and eyes with normal choroidal permeability with IVR. To assess the effectiveness of IVR, we used a new method of studying the choroid by spectral-domain optical coherence tomography (OCT) called enhanced depth imaging OCT (EDI-OCT).

## Methods

The procedures used in this study were approval by the Institutional Review Board of the Kagoshima University Graduate School of Medical and Dental Sciences, Kagoshima, Japan. The procedures conformed to the tenets of the 1989 Declaration of Helsinki.

We reviewed the medical records of patients diagnosed with PCV who were examined between September 2010 and December, 2011. The clinical diagnosis of PCV was based on the presence of polypoidal choroidal vessels in the ICGA images. All patients underwent a comprehensive ophthalmological examination including measurements of the best-corrected visual acuity (BCVA), intraocular pressure measurements, slit-lamp biomicroscopy, dilated funduscopic examination, and fundus photography. Fluorescein angiography (FA), indocyanine green angiography (ICGA), and EDI-OCT were performed with the Heidelberg Spectralis® OCT and HRA instruments (Heidelberg Engineering, Heidelberg, Germany). The clinical diagnosis of PCV was based on the presence of polypoidal choroidal vessels in the ICGA images. The inclusion criteria were: presence of subfoveal serous retinal detachment or serous pigment epithelial detachment (PED) or both at the fovea; absence of hemorrhage and fibrin at the fovea; absence of an obvious fibrovascular membrane at the fovea; no previous PDT or IVR; and active leakage on FA caused by the PCV including choroidal vascular hyperpermeability and not from other diseases.

The patients were divided into a hyperpermeability group (HP group) and a normal choroidal permeability group (NP group) based on the ICGA finding of choroidal hyperfluorescence in the midphase of ICGA about 10 minutes after ICG injection. The classification was made by 3 masked graders independently (NA, SS, HO). The eyes with choroidal hyperpermeability, i.e., choroidal vessels with leakage of the ICG dye, were classified according to an earlier study and were placed in the HP group [4]. Three graders classified each case independently without any information about the eye including the choroidal thickness. When there was a disagreement, the classification supported by two graders was chosen as the final classification. All cases were classifiable. Because the classification was subjective, the inter-grader agreement rate was evaluated by the Fleiss’s kappa measure to assess the reliability of the agreements.

The central choroidal thickness and the central foveal thickness (CFT) at the baseline and 7 days after the IVR were measured in the EDI-OCT images with a follow-up program as we have described in detail [21]. Following each examination, the best image was projected onto a computer screen and evaluated by 2 independent masked graders (NA and MS). Measurements of the choroidal thickness (rating) was done in a masked fashion by these raters with no information of the eyes. For inter-rater comparisons, each image was measured by two raters with no information of eyes or the results by the other rater. These two values were used for the analysis. The average of the results by two raters was used for the analysis of choroidal thickness. All of the images were obtained using the eye-tracking system. The OCT Star (enhanced depth imaging) protocol with 768 A scan/B scan and averaged 100 scans was used. The central choroidal thickness was measured from the border of Bruch’s membrane to the inner border of the sclera. The CFT was measured between the inner border of the neurosensory retina and Bruch’s membrane including any PED. The percentage reduction in the CFT was calculated by the following formula.

Percentage reduction rate (%) = 100 × (1-[post-IVR CFT/baseline CFT]).

The best-corrected visual acuity (BCVA) was measured with a Japanese standard decimal visual chart at the baseline and 7 days after the IVR. The decimal acuity was converted to the logarithm of the minimal angle of resolution (logMAR) units for the statistical analyses. The age, sex, treated eye, abnormal findings, e.g., RPE atrophy, drusen, and PED in the contralateral eye, lens status, and refractive error were also obtained from the medical charts.

All statistical analyses were performed with the statistical programming language R (ver. 2.11.1, The R Foundation for Statistical Computing, Vienna, Austria). The inter-grader agreement was compared using Fleiss’ kappa coefficient. The Mann–Whitney U test and Chi-square test were used to determine the significance of differences of 2 groups. The inter-rater correlation coefficient using a two-way mixed effects model for measurements of absolute agreement were computed. Multivariate regression (stepwise) analysis was performed on age, sex, permeability, CCT, laterality, baseline CMT, lens status, refraction, and baseline logMAR BCVA. A *P* value < .05 was considered statistically significant. A Fleiss’ kappa value of 0.01 to 0.20 was considered to be a slight agreement, 0.21 to 0.40 a fair agreement, 0.41 to 0.60 as moderate agreement, 0.61 to 0.80 as substantial agreement, and 0.81 to 1.0 as very good agreement as reported [22]. A kappa value <0 was considered to be no agreement.

## Results

Fifty-seven eyes without previous treatment were diagnosed with PCV, and 42 of these eyes of 42 patients met the inclusion criteria. Among the 15 excluded eyes, ten eyes were excluded because of the presence of hemorrhage or fibrin at the fovea, 3 eyes because of the presence of a fibrovascular membrane, and 2 eyes because of a lack of clear OCT images.

In the classification of the 42 eyes, 38 eyes were unanimously agreed to by the three raters and 4 were not unanimous. For the agreement between raters, intraclass correlation coefficient expressed as Fleiss’ kappa was 0.95 (*P* < .0001, 95% confidential interval 0.92 – 0.97), which is an almost perfect agreement [22].

**Figure 1:**
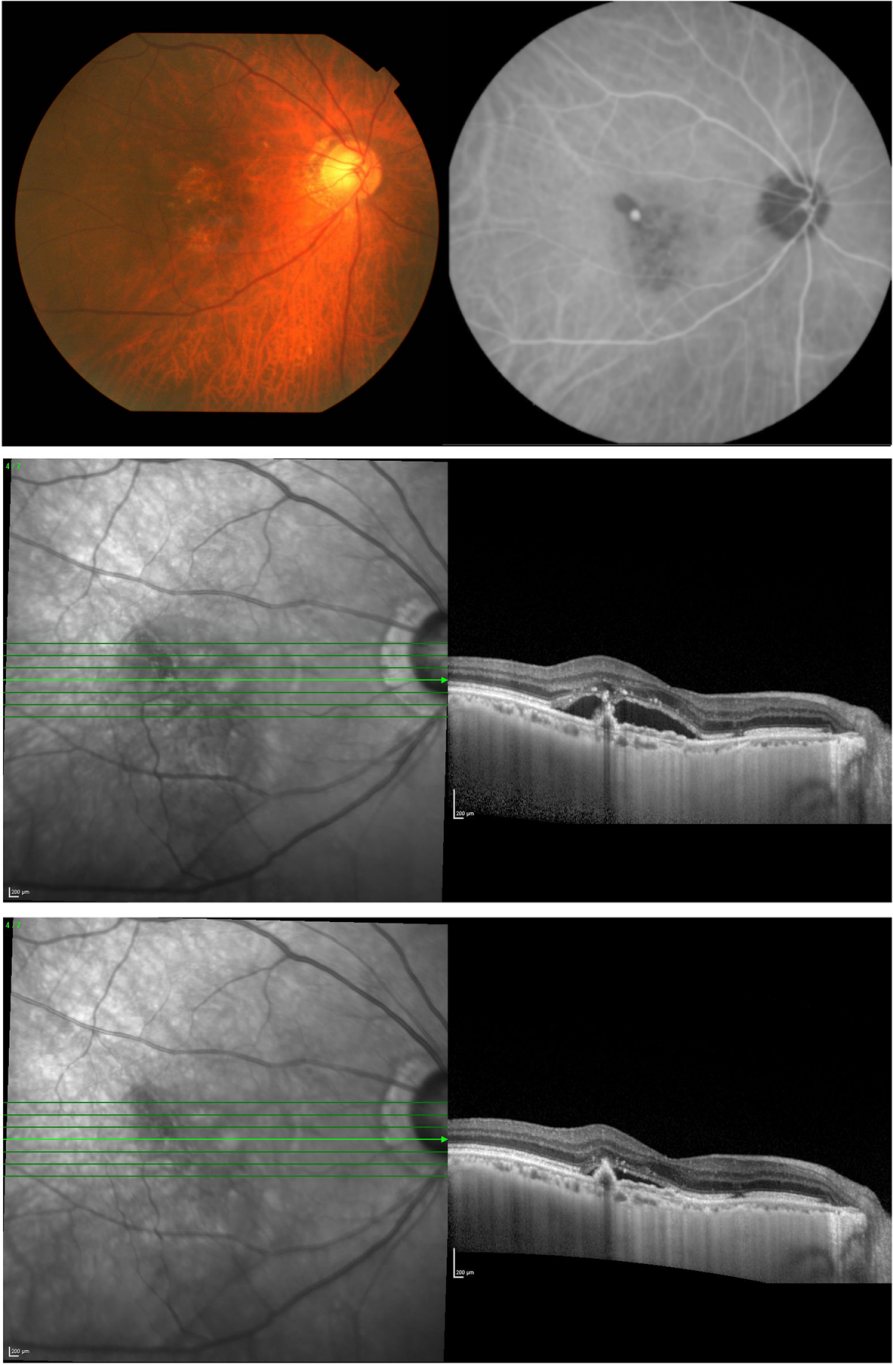
**Fundus photograph, indocyanine green angiogram, and EDI-OCT images of an eye with normal choroidal permeability (NP group).** An orange-colored polypoidal lesion can be seen adjacent to the foveal area (top left). Indocyanine green angiogram shows a polypoidal vascular lesions (top right). Choroidal hyperpermeability is not apparent throughout the examination (top right). EDI-OCT shows an elevation of the RPE layer and serous retinal detachment (middle). Seven days after intravitreal ranibizumab (IVR), the serous retinal detachment is reduced (bottom).

**Figure 2:**
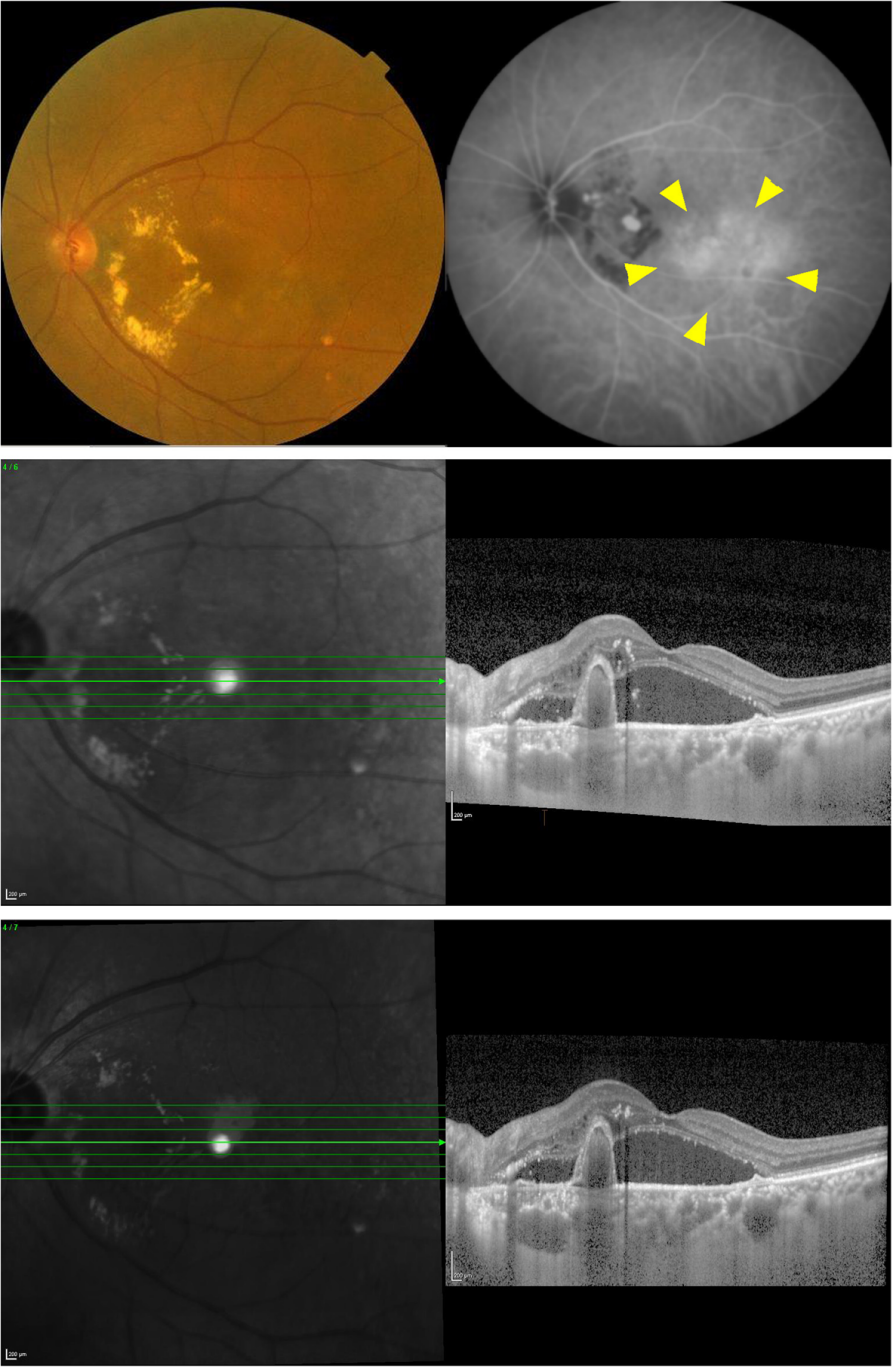
**Fundus photograph, indocyanine green angiogram, and EDI-OCT image of an eye with PCV with choroidal hyperpermeability (HP group).** An orange-colored polypoidal lesion surrounded by hard exudates can be seen between the optic disc and macular area (top left). Indocyanine green angiogram shows several vascular polypoidal lesions. Choroidal hyperpermeability (arrow heads) can be seen (top right). EDI-OCT image shows an elevation of the RPE layer and thick choroid. Serous retinal detachment is also seen (middle). Seven days after IVR, the elevated RPE layer, serous retinal detachment, and thickened choroid did not change significantly (bottom).

In the end, 21 eyes were placed in the HP group and 21 in the NP group. The baseline demographics of each group are summarized in Table 1. The fundus photographs and the ICGA and OCT findings of a representative case from the NP group are shown in Figure 1, and those of a representative case from the HP group are shown in Figure 2.

**Table 1:** Characteristics of the patients

	**HP group (n = 21)**	**NP group (n = 21)**	***P *value**
Gender (M/F)	16 / 5	13 / 8	0.317†
Age (y.o.)	64.5 ± 6.8	72.4 ± 9.4	0.008‡*
Treated eye (R/L)	9 / 12	8 / 13	0.753†
Contralateral eye involvement (+/−)	3 / 13	7 / 14	0.277†
Lens status (IOL eye)	2	0	0.147†
Refraction (D)	0.14 ± 1.82	0.55 ± 2.10	0.458‡

†Chi-square test. ‡Mann–Whitney U test. *Significant value.

*D* Diopter, *F* Female, *HP* Hyperpermeability, *IOL* Intraocular lens, *M* Male, *NP* Normal permeability.

The mean age of the HP group was 64.5 ± 6.8 years, and 16 (76.2 %) of them were men. The mean age of the NP group was significantly older at 72.4 ± 9.4 years (*P* = .008), and 13 (61.9 %) of them were men. The difference in the incidence of choroidal vascular hyperpermeability between men and women was not significant. In addition, the incidence of the contralateral eye involvement, laterality of the treated eye, lens status, and refractive at the baseline error were not significantly different between the HP and the NP groups. The BCVA at the baseline and at 7 days after IVR were not significantly different between the two groups, and the IVR did not improve the BCVA for at least 7 days after the treatment in both groups.

The inter-rater agreement of the choroidal thickness was very high with a coefficient of variance 0.932. The intraclass correlation coefficient had a 95% confidence interval of 0.887 to 0.964.

The mean central choroidal thickness at the baseline was significantly thicker in the HP group than in the NP group (378.6 ± 92.5 μm vs 192.5 ± 58.1 μm; *P* < .001). A significant reduction of the mean CFT between baseline and post-IVR was found in both groups. In addition, the percentage reduction of the CFT in the HP group was significantly less than in the NP group (14.0 ± 7.5% vs 20.4 ± 8.7%; *P* = .013; Table 2). Additionally, multivariate regression (stepwise) analysis was performed on age, sex, permeability, CCT, laterality, baseline CMT, lens status, refraction, and baseline logMAR BCVA. This allowed us to obtain the variables for the regression equations including permeability (HP or NP) and laterality. As a result, R = 0.478 with a *P*-value of 0.009 for permeability and 0.042 for laterality. Thus, the FA permeability of choroid was still significant for the reduction of CFT.

**Table 2:** Comparison of choroidal/foveal thickness and visual acuity between two groups

	**HP group (n = 21)**	**NP group (n = 21)**	***P *value†**
Central choroidal thickness (μm)	378.6 ± 92.5	192.5 ± 58.1	<.001*
Baseline CFT (μm)	424 ± 147	461 ± 174	0.554
Post CFT (μm)	363 ± 131	366 ± 140	0.821
Reduction rate of CFT (%)	14.0 ± 7.5	20.4 ± 8.7	0.013*
Baseline VA	0.281 ± 0.269	0.365 ± 0.377	0.725
Post VA	0.274 ± 0.250	0.368 ± 0.392	0.715

†Mann–Whitney U test. *Significant value. *CFT* Central foveal thickness, *HP* Hyperpermeability, *NP* Normal permeability, *VA* Visual acuity.

## Discussion

Our ICGA results showed that the choroid of about one-half of the eyes with PCV were hyperpermeable to indocyanine green. In addition, the choroid of these eyes was significantly thicker than that of eyes with normal permeability which is in agreement with earlier reports [15,16]. This is important because these eyes responded less well to IVR, viz., the percentage reduction in the CFT after IVR in these eyes was significantly less than that in eyes with normal permeability. This would suggest that the CFT in these eyes was less influenced by an up-regulation of VEGF than the eyes with normal permeability.

Choroidal vascular hyperpermeability is also present in eyes with CSC, and the hyperpermeability of choroid has been suggested to be the pathophysiological basis of CSC [23-25]. Hyperpermeable choroidal vessels increase the tissue hydrostatic pressure, which overpowers the barrier functions of the RPE leading to serous retinal detachments [26]. There are various factors that cause the hyperpermeability of the choroid in CSC such as stasis, ischemia, inflammation, and other related factors [27]. At present, PDT is regarded to be the standard treatment for CSC [27]. Although the real mechanism of how PDT resolves the CSC has not been fully determined, it is assumed that PDT induces choroidal vascular remodeling with thinning of the choroid [28]. This effect was assumed to also occur in eyes with PCV by the frequent disappearance of the polypoidal lesions after PDT treatment [7].

On the other hand, anti-VEGF agents are not considered first-line treatments for CSC. Several small trials have yielded suggestive results, although their effectiveness has not been confirmed. Bae et al. showed in a small randomized trials that half-fluence PDT may be superior to anti-VEGF agent as a treatment for CSC [29]. Anti-VEGF agents are highly effective in reducing subretinal fluid, reducing extravasation from retinal vessels, and neovascularization, but they may not be sufficient to reduce the hyperpermeability of the choroid in eyes with CSC and PCV. The similarities between the PCV eyes with hyperpermeability and CSC eyes suggest that they may share features relating to their pathogenesis and pathophysiology [6,10,11,27].

Lim et al. reported that the responsiveness to bevacizumab as shown by the resolution of the subretinal fluid in eyes with CSC was better in eyes with than without hyperpermeability. Their results are just the opposite of our results [23]. However, Koizumi et al. recently reported that the responsiveness to ranibizumab, expressed as resolution of retinal fluid in eyes with PCV, was worse in eyes with than without hyperpermeability which is consistent with our results [30]. Lim et al. studied eyes with CSC and not with PCV. Although CSC and PCV have certain similarities, they are not the same disease. Additionally, the definition of hyperpermeability in CSC may not be the same as that of PCV. These factors might explain the different response patterns to the treatment between the reports.

In this past decade, new treatments have been introduced for exudative AMD [1,6]. PDT alone or IVR alone or a combination of the two have been used to treat AMD with sub- or juxta-foveal lesions but the outcomes of these therapies have not been consistent. PDT was not as effective for eyes with typical AMD but relatively better for eyes with PCV [6,7]. In contrast, anti-VEGF therapy was more effective for eyes with typical AMD, but its effectiveness on eyes with PCV was clearly inferior to that on eyes with typical AMD [6,11]. However, it has not been determined whether the type of treatment for cases of typical AMD should be different from that of cases of PCV.

Our results indicated that the choroidal thickness might serve as an indicator of the responsiveness of an eye to anti-VEGF treatment. For example, it is known that the choroid is very thin in eyes with pathological myopia [31]. Our findings suggest that IVR would be effective in reducing the CFT in these eyes. This has been confirmed by a study that showed that anti-VEGF treatment for choroidal neovascularizations secondary to pathological myopia was effective [32]. Therefore, the association between choroidal thickness and the efficacy of IVR should be determined for other types of exudative AMDs.

There have been recent reports on the results of combined PDT and anti-VEGF antibodies to treat PCV. Lai et al. reported that IVR resulted in a stabilization of vision in patients with symptomatic PCV. However, combined IVR and PDT appeared to be more effective in causing a complete regression of the polypoidal lesions in ICGA compared with IVR monotherapy [33]. Others have shown the comparatively good results for maintaining the visual acuity [34,35]. Because there is no large scaled analysis of anti-VEGF monotherapy and combination therapy, the results are not conclusive. Considering our cases with PCV that are non-responsive to IVR, it might be meaningful to select specific cases requiring PDT rather than IVR (with or without PDT). The present finding would be helpful for that.

More importantly, there is another consequence of our findings. Treatments based on antibody-drug technology including IVR could produce a so-called knock-out (KO) condition in human eyes. So far, this condition has been accomplished only in animals because of ethical reason, however the results from animal studies do not necessarily reflect those of human disease. The present method and interpretation, called interventional immunology, might be suitable for ocular diseases because the eye is a relatively closed system and is less affected by the systemic circulation [18,19]. Although molecules injected into the vitreous can appear in systemic circulation, its effect is less than after an intravenous injection as is performed in cancer therapy.

Currently, PCV is classified as being a type of AMD, but recent genetic analysis and angiographic studies of Japanese populations showed that there are some subtypes of PCV [36-38]. Our findings showed the role of VEGF in the disease process of PCV, and these eyes may be a sublclass of PCV eyes that are not related to VEGF pathology.

From the early days of PCV study, it was asked whether PCV originated from abnormal choroidal vessels or was a variant of type 1 CNV [4-6]. This issue might be related to the responsiveness to IVR. Further studies are needed. To answer this question, further studies with more advanced method such as simultaneous ICGA and eye-tracked SD-OCT would be helpful in obtaining an answer [39].

The limitations of this study are the relatively small number of patients studied and the short follow-up period. Because we limited our patients to those that had neither apparent hemorrhage nor fibrin but had pure serous exudation at the fovea, the responsiveness to IVR could be detected clearly even 7 days after the IVR. In addition, we showed the short term effects of anti-VEGF drug. Because VEGF is not only an angiogenic factor but also a pluripotent inflammatory factor, the long term *in vivo* results can be affected by various factors other than VEGF [40]. Thus, the short term results would be more meaningful for evaluating the direct effect of VEGF on PCV pathology. However, this does not necessarily mean that the long term results might not be the same. Even though, the classification was done in a masked fashion and high inter-rater agreement was obtained as confirmed by a Fleiss’s kappa of 0.95, this was not a perfectly objective system and this method is not equally applicable to every clinical setting. In addition, this was not a prospective randomized study but a retrospective study. As was described in the Results section, eyes with hazy media or poor OCT images were not included in this study. Thus, there is a potential selection bias in this study. These issues should be remembered when interpreting our results.

## Conclusions

In conclusion, some of the eyes with PCV had choroidal hyperpermeability, and these eyes were less responsive to IVR. This would suggest that the choroidal permeability in eyes with PCV is less dependent on VEGF-related pathology. For these eyes, combination therapy might be more suitable than IVR monotherapy. Evaluation of the choroidal thickness may be a way of predicting the responsiveness of eyes with PCV to IVR. Future studies need to be performed to determine whether this relationship is present in other types of exudative AMDs.

## Competing interests

The authors declare that they have no competing interests.

## Authors’ contributions

SS and TS were involved in the design and conduct of the study. Collection and management of the data were done by HO, NY, YY, YK, and AO, while analysis and interpretation of the data were performed by SS, TS, and NA. TY performed the detailed statistical analysis. Preparation of the first draft of the manuscript was done by TS and review and approval of the manuscript was performed by NA and TS. All authors read and approved the final manuscript.

## Pre-publication history

The pre-publication history for this paper can be accessed here:

http://www.biomedcentral.com/1471-2415/13/43/prepub
